# Adolescents and Young Adults with Cancer and the Desire for Parenthood—A Legal View from a Swiss Perspective in Consideration of the Relevance of Cancer Support Organizations

**DOI:** 10.3390/curroncol30120736

**Published:** 2023-11-27

**Authors:** Isabel Baur, Sina Staudinger, Ariana Aebi

**Affiliations:** 1 Competence Center of Medicine-Ethics-Law Helvetiae, University of Zurich, 8032 Zurich, Switzerland; ariana.aebi@ius.uzh.ch; 2 The Association “AYA Cancer Support CH”, 8041 Zurich, Switzerland; sina.staudinger@ius.uzh.ch; 3 Committee for Protection against Sexual Harassment, University of Zurich, 8032 Zurich, Switzerland

**Keywords:** AYA oncology, treatment, models of care, long-term follow-up, survivorship, fertility, cancer association, peer group

## Abstract

This commentary focuses on the challenges and possibilities that adolescents and young adults with cancer (AYA) desiring parenthood face under Swiss law. The regulation of reproductive medicine procedures is stricter in Switzerland than in some other countries. Health insurance is compulsory, but the interventions that are covered are in constant flux. Recent changes pertain to the possibilities of future AYA parenthood and keeping up to date with practical and legal ramifications is taxing even for health professionals. AYA facing treatment decisions are uniquely vulnerable and dependent on comprehensive, clear, current, and country-specific information regarding risks and options pertaining to their fertility. This commentary provides a short overview of the Swiss legal framework related to reproductive medicine, highlighting its access restrictions and prohibitions, as well as recent changes. While the importance of patient, peer, caregiver, and interest groups supporting people affected by health conditions has long been recognized in many countries, an AYA organization was only recently established in Switzerland. Such organizations are vital for providing accurate, country-specific information and support, while individualized medical guidance, informed by the most current legal framework and its consequences, remains essential in addressing AYAs’ specific needs in connection with the desire to have children.

## 1. Introduction

Globally, approximately 1.2 million adolescents and young adults aged 15 to 39 years are diagnosed with cancer every year [1,2]. There is currently no consistent definition of the age range of AYA. According to common definitions, adulthood begins at around 20 years of age and young adulthood ends—depending on the point of view—at 24, 35 or 39 years. Since AYA have only recently been recognized as a distinct patient group in Switzerland, and since there is still no research in this area in Switzerland taking into account legal aspects, the authors have chosen this patient group. In the context of challenges regarding the fertility of AYA, an age range up to 39 years is justified [3]. In the past decade, AYA have become recognized as a vulnerable patient group [4]. In Switzerland—where AYA have long been unrecognized as a distinct patient group—calculations have shown that approximately 1770 AYA are newly diagnosed with cancer each year [5]. Due to progress in cancer treatment, over 80% of AYA diagnosed with cancer survive beyond 5 years, which leads to a continual increase in the number of AYA survivors who have the potential to continue living for decades [2,4]. This requires that the health system guarantees access to treatment and follow-up care in various medical and psychosocial areas beyond oncology [3].

The need to regard AYA as a distinct patient group affected by a broad spectrum of cancers stems from their life stage, their specific psychosocial needs, and the effects as well as long-term consequences of the cancers and their treatments, as well as the potential years of life lost [2]. AYA are at a stage in life where they may be very conscious of their body and appearance and would, under normal circumstances, develop a positive body image, start leaving home and establish independence, increase contact with peers, and start dating or making important career and family decisions [2,6]. It is essential to take into account that a cancer diagnosis—especially concerning younger AYA—affects not a mature but a maturing personality, in a developmentally vulnerable period, and still in the process of finding their identity [7,8]. The diagnosis comes at a time when under normal circumstances the fear of dying would have no place, with the diagnosis interfering with life overall and future planning [9]. Long-term and late effects among AYA survivors cover a range of physical issues (due to changes in appearance), secondary malignancies (for example, due to radiotherapy), cardiovascular diseases, endocrine dysfunctions (such as thyroid dysfunction or diabetes), neurocognitive deficits, impaired fertility, sexual dysfunction, body disfigurement, a lower level of physical functioning, and psychological and social issues (due to disruptions in social life, increased dependence or a premature confrontation with mortality), as well as challenges in finances and career [2,6]. In connection with the desire for parenthood, it should be mentioned that half of all male childhood cancer survivors suffer from infertility as a late consequence of treatment [10]. The lived experience of young survivors regarding decisions about fertility and parenthood has received insufficient attention in the literature to date. There is a clear unmet need to provide age-appropriate information regarding fertility and parenthood options [11]. Furthermore, an AYA’s cancer diagnosis may also affect relatives, partners or caregivers, as well as potential future partners [2,12].

As legal volunteers for the non-profit organization “AYA Cancer Support CH”, the authors have repeatedly been confronted with the legal challenges associated with survivors’ desire to have children. This commentary provides a broad overview of the current practice in Switzerland from a legal perspective. In Switzerland, medical insurance is compulsory and covers interventions that meet certain criteria, such as cost-effectiveness, the definition of which is a matter of ongoing debate. Medically assisted reproduction is more strictly regulated in Switzerland than in other countries, which has implications for AYA, requiring measures for human reproduction and fertility preservation due to the impact of chemotherapy. This paper answers questions such as the following: To what extent are human reproduction measures for AYA possible, be it legally or practically, in Switzerland? How are costs covered, e.g., in connection with egg storage? Due to cancer support organizations becoming increasingly relevant in Switzerland, where peer, family, and caregiver support groups have been less common in the past than they have been in other countries, this commentary aims to bridge the gap between legal practice and constraints and the role of non-profit organizations to support AYA with lived experiences of cancer and their partners considering a current or future wish to become a parent.

## 2. Materials and Methods

This commentary is based on a systematic literature search and the personal experiences of the three authors and co-founders of the AYA organization “AYA Cancer Support CH”. International AYA-related studies, topic-specific literature, and Swiss legal opinion publications as well as Swiss case law and court rulings were used, taking into account current developments and innovations in legislation. The choice of literature was at the discretion of the authors. Most of the literature analyzed is relevant to the field and scientifically based. In isolated cases, e.g., with regard to supportive measures, sources relevant to practice were also used.

## 3. AYA, Fertility and Childbearing

### 3.1. Introduction

Involuntary childlessness—for a variety of reasons—may place a considerable burden on people of all genders [13]. Although cancer therapy can severely limit fertility, many AYA do not consider dealing with a possible future desire to have children when confronted with a cancer diagnosis. Rather, the disease and its treatment take center stage and disrupt the planned life course. In the general public in Switzerland, the mean age for giving birth for the first time is 31 for the person carrying the child [14]. This points to the fact that the discussion about having children usually follows at a later stage in life.

For people affected by cancer at a later stage in life, the desire to have children may already be present at the time of diagnosis or efforts may already have been made to become pregnant. At this stage of life, the topics of the desire to have children and fertility already lead to considerations of whether cryopreservation should be considered at the time of diagnosis. Fertility can be limited by impending cancer therapy, which can lead to full infertility. However, this is not always the case, as different therapies entail different consequences in terms of fertility. Nevertheless, with a view to the future, the retrieval and cryopreservation of oocytes or sperm early on may greatly improve the chance of preserving fertility [15].

In addition to medical questions and decisions in the context of a cancer diagnosis, for a physician, it would be particularly important to also discuss the topics of “sexuality”, “fertility” and “desire for children” with young patients. Sexuality and fertility are often dismissed in society and problems in this area tend to receive little attention. Nevertheless, it is of utmost importance that physicians address the issues, outline the various consequences, and provide information so that a conscious decision can be made for or against fertility-preserving measures [16]. The inclusion of various options is of great importance in this decision-making process. This is because even if a therapy is to be used and the treatment is expected to have little or no effect on fertility, scenarios with less favorable or different outcomes need to be considered up front. For example, it may be necessary to switch therapies due to treatment resistance, cancer progression, or the intolerability of side-effects. It is the duty of the physician to inform young patients of the choices and potential consequences. These decisions and burdens should not be underestimated in the overall process of treatment. The issue of fertility in AYA requires collaboration and cooperation among specialists in oncology, reproductive medicine, and pediatrics, as appropriate. Certain considerations may be obvious and self-evident to specialists; however, they must be communicated and discussed with the patient in concrete terms in order to enable the patient to grant truly comprehensive informed consent [15].

### 3.2. Legal Framework in Switzerland

#### 3.2.1. Current Situation in Switzerland

In Switzerland, approximately 6200 women made use of reproductive medicine options (in vitro methods) in 2020 [17]. The common reasons why couples remain involuntarily childless and make use of medically assisted reproduction vary. For example, the first pregnancy resulting in childbirth increasingly occurs in those over 35 years old because the reconciliation of work and family life is difficult. Another reason can be found in genetic diseases that could be transmitted to the newborn [13]. These reasons may also apply to AYA. However, AYA face additional challenges. In the following sections, the legal situation in Switzerland with regard to the possible use of medical reproductive procedures is outlined.

**Figure 1:**
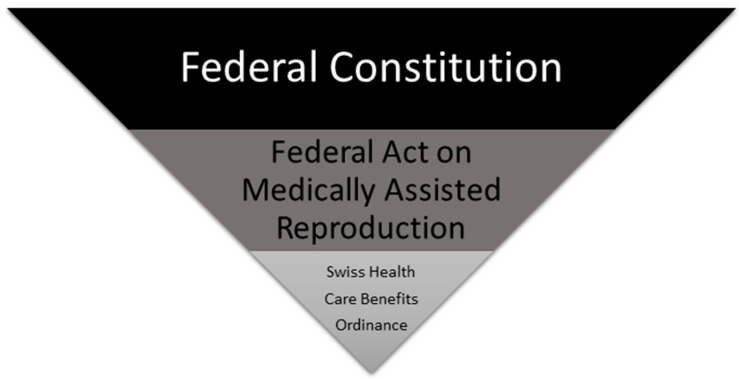
Visualization of the hierarchy levels of the different laws.

The following figure (Figure 1) provides an initial overview of the relevant laws in Switzerland:

#### 3.2.2. Federal Constitution and Legislative Level

The Federal Constitution (FC) [18] is the constitution of the Swiss Confederation and is hierarchically at the highest level of the Swiss legal system. Laws or ordinances of the Confederation as well as the cantons and the communes are subordinate to the Federal Constitution [19]. The desire to have children is recognized as an elementary element of personality development and is protected by the fundamental right of personal freedom, which is protected by the Confederation in Art. 10 para. 2. In concrete terms, this protection means that, on the one hand, access to methods of reproductive medicine is guaranteed, and on the other hand, human beings are to be protected from abuses of reproductive medicine. Human dignity in dealing with human germinal and genetic material takes precedence at all times. The Confederation has the competence to regulate the handling of human germinal and genetic material (Art. 119 para. 2 FC). It is prohibited to use medically assisted reproduction in order to conduct research or to induce certain characteristics in a child (Art. 119 para. 2 lit. c FC).

Furthermore, the Constitution forms the basis of specific substantive requirements for the use of reproductive medicine, which are specified by the Federal Act on Medically Assisted Reproduction (RMA) [20].

#### 3.2.3. Access Restrictions and Prohibitions

Both the FC and the RMA contain restrictions and limitations on access. The declared aim of the restrictions is to ensure the best interests of the child. This is because medically assisted reproduction may only be used if the best interests of the child, as the overriding good, are safeguarded (Art. 3 para. 1 RMA). The scope, meaning and understanding of what the best interests of the child entail are not legally defined [13] and are subject to social views and developments.

In order to do justice to the best interests of the child, various prohibitions are contained in both the FC and the RMA, which are handled less restrictively in other countries. Of particular relevance for AYA is that both embryo donation and all forms of surrogacy are prohibited in Switzerland (Art. 119 para. 2 lit. d FC). Furthermore, according to Art. 4 RMA, egg donation is prohibited. However, artificial insemination, in vitro fertilization with embryo transfer, and gamete transfer are generally permitted (Art. 2 lit. a RMA, Art. 5 and Art. 5b RMA). In particular, surrogacy and egg donation are the subject of a lively social and professional debate [13,21]. Especially for young female AYA, the prohibition of surrogacy has implications if there is a desire to have children and the woman’s fertility has become limited or fully impaired. In this case, it is not permitted for another person to carry a child by means of egg donation or with the egg of the AYA concerned.

In addition to the prohibitions described above, Swiss law also imposes various restrictions on access to reproductive medical procedures. The procedures are reserved for couples with whom a future child is deemed to be able to establish a parent–child relationship in accordance with the Swiss Civil Code, and who, based on their age and personal circumstances, are capable of caring for such a future child until it reaches the age of majority (Art. 3 para. 2 lit. a and b RMA). If this is considered to be established, the reproductive procedure must serve to overcome the infertility of the couple, if other methods of treatment do not lead to the desired outcome or if there is a risk of transmission of a serious disease (Art. 5 lit a and b RMA). Only married heterosexual and—only since 1 July 2022—homosexual couples can make use of sperm donation (Art. 3 para. 3 RMA).

### 3.3. AYA and the Desire to Have Children

After the preceding explanations, the question arises as to what extent AYA can make use of reproductive medicine in Switzerland. According to the current law, germ cells may be preserved (Art. 15 RMA). Progress in reproductive medicine has led to a continued improvement in the possibilities of preserving germ cells. From a legal point of view, germ cells can in principle be preserved for five years and, at the request of the person concerned, the preservation may be extended by a maximum of five years (Art. 15 para. 1 RMA). A longer preservation period is also possible if a medical treatment is carried out which the person concerned must undergo, or if he/she must perform an activity that may lead to infertility or damage to his/her genetic material (Art. 15 para. 2 RMA). So-called cryopreservation refers to the method by which vital cells (male or female) or fertilized oocytes or embryos can be frozen [22]. There are different methods, especially “slow freezing” and “vitrification”; while the choice of the method is considered less decisive, the factors of the time of collection and the time of possible use of the frozen cells are considered crucial. Even the duration of storage is considered less relevant. From a technical point of view, the freezing and thawing process plays a more important part [22].

The storage of germ cells is agreed upon with a contract: a so-called cryo-contract (deposit contract according to Art. 472 Code of Obligations [23]). In this contract, the custodian is charged with the safekeeping of a movable object (unfertilized extracorporeal germ cells) entrusted to him or her by the depositor. Use without the depositor’s consent is prohibited. The contract is usually renewed annually [22]. An annual storage fee is payable for the service. In addition, there are costs for ovarian stimulation and egg retrieval. Compulsory health insurance does not usually cover these costs and so storage must be self-financed by the person concerned. However, since July 2019, there has been an exception for fertility-preserving measures for men and women up to 40 years of age suffering from cancer (Art. 1 Swiss Health Care Benefits Ordinance (KLV) [24] in connection with Annex 1 No. 3 KLV). This provision may be considered an important step in the perception and concrete support of AYA in Switzerland. The exception indicates that the legislator and the public are developing an awareness of the AYA patient group as well as their specific needs.

For AYA with the desire to father offspring through reproductive medicine, the provision of Art. 15 RMA is of particular importance. On the one hand, a reproductive procedure can be used to circumvent infertility caused by cancer therapy. Medical treatments leading to infertility, surgical interventions, radiological treatments, chemotherapies or potentially fertility-toxic medication are to be thought of [22]. On the other hand, there are also cancers that can be transmitted to offspring. In these cases, reproductive medicine can avert this risk to the extent that the germ cells are specifically selected in an in vitro procedure to avoid this risk (Art. 5a RMA). Regarding future medical treatment leading to damage to the genetic material or to a permanent loss of fertility, it is possible to preserve germ cells beyond the ten-year storage period.

In addition to the medical support options described above, it should be mentioned that the possibility of adoption must also be considered. The legal requirements for adoption are also regulated nationally and are considered strict in Switzerland in comparison to other countries (Art. 264 ff. Swiss Civil Code [25]). A detailed explanation of the legal basis with regard to adoption would go beyond the scope of this commentary.

### 3.4. AYA Organisations

The above-mentioned legal and medical challenges regarding parenthood after a cancer diagnosis can be quite complex, and a lack of knowledge about possible measures and possibilities could even lead to an unfulfilled desire for parenthood. In order to counteract this lack of information as well as other possible long-term consequences of cancer, support by organizations tailored to the needs of AYA during and after treatment as well as for their relatives, partners, friends and caregivers is essential. Psychosocial and behavioral interventions may assist AYA in returning to their social roles as a parent, spouse, student, worker or friend, and provide support with finding specific and relevant cancer-related information, as well as measures for specific problems, such as coping with the future, possible impaired fertility, fatigue, fear of relapses, or challenges concerning the resumption of work or education [6,26]. Peer support among AYA in dealing with long-term consequences, sharing their lived experience in an age-appropriate way, and talking to someone in the same situation about specific problems is vital [6,27]. AYA have to face different questions due to their stage in life, such as the following: How much should they tell new acquaintances, including an employer or someone they are newly dating, about their illness or long-term impacts [6]? Where can they find medical and psychological support once their treatment ends [26]? How should they deal with friends lost due to cancer [26]? Does cancer during pregnancy effect the unborn child? What happens during breastfeeding while having breast cancer? These are all questions where AYA could benefit from the experiences of other AYA who have already experienced these situations.

There are many different organizations worldwide for AYA. Table 1 is an attempt to categorize some of these organizations, although the list is in no way exhaustive and certain organizations may include services that would also make them fall in more than one category:

As shown by the incredible number of users on the two largest web-based social media platforms [47], Stop Cancer (with over 30,000 followers on Facebook) [48] and Stupid Cancer (with almost 315,000 followers on Facebook) [49], organizations that utilize digital measures have gained massive relevance when it comes to supporting AYA. Through using technologies that AYA as digital natives are familiar with (Facebook, Youtube, etc.). Web-based social networking websites help AYA to connect with each other and therefore give an opportunity for peer involvement as well as social support through peer interaction, where AYA can exchange the above-mentioned fears or social issues [6,26]. Social digital media platforms allow a multi-perspective exchange that can cover topics ranging from social security to clinical information, for example, on side effects and personal experiences, and can transition into a mentoring program, where survivors support newly diagnosed AYA [47]. The great advantage of digital and virtual measures, be it to connect AYA with each other or also to provide professional, cancer-specific information, is that the measures are suitable for AYA despite the geographical range, therefore reducing expensive as well as time- and energy-consuming trips to hospitals, self-help groups, etc. [26]. In addition, digital measures are constantly available and convenient, while the informal nature of the measures could make AYA feel less stigmatized and give them the opportunity to maintain their anonymity [47].

With regard to the desire for parenthood, such organizations could play a central role in providing further information in order for AYA to be informed about the medical and legal options available to them. Additionally, such organizations could also promote the exchange with other AYA who are in the same situation or who have been able to successfully fulfill their desire for parenthood.

Looking at the table above on the various existing AYA organizations, it is disconcerting that until recently, Switzerland did not have its own AYA organization, neither as a digital nor as a local organization. This could be due to the fact that AYA have only recently been perceived as a distinct patient group, or maybe perhaps also due to the state’s social security net, which in Switzerland might be stronger than that in some other countries. However, the situation changed in 2019, when the Competence Center for Medicine-Ethics-Law Helvetiae [50] together with the PhD Program Biomedical Ethics and Law/Law Track [51], both initiatives of the University of Zurich in cooperation with All.Can Switzerland [52], launched the Project “Fresh Ideas for Cancer Care”, in which one group of PhD students received the task to find innovative cancer support programs for teenagers and young adults [53]. This sub-project ended with the realization that there are no AYA organizations in Switzerland and that AYA-specific services [54] are very fragmented regionally but also among organizations [9]. The sub-project did not stop at this result, but the scientific results gained from this research were used to create the platform “ayacancersupport.ch”, which brings together the various support services as well as events specific to AYA in Switzerland. By now, the offer of web-based social networking possibilities has also been extended to various social media channels to enable AYA to connect with each other [55,56]. To give AYA a voice in Switzerland, to promote the peer community and thus also the exchange, as well as to contribute to their needs receiving much-needed political attention, the authors founded the non-governmentally financed non-profit AYA association “AYA Cancer Support CH” with the platform “ayacancersupport.ch”. AYA are in a position of particular vulnerability, and are generally ill-equipped to proactively bring up questions of possible future parenthood and engage in patient-led or collaborative decision-making with their treatment team, who may themselves find aspects such as the legal frameworks overwhelming [57,58]. The availability of accessible, clear and age-appropriately worded, up-to-date information about the Swiss legal framework and practice pertaining to AYA parenthood is key to empowering AYA in their decision making and, if the AYA desire this, enabling their partner’s and family’s support [16,58,59,60,61]. It is the authors’ aim as legal scholars to help bridge this knowledge gap.

## 4. Conclusions

Swiss law grants access to medically assisted reproduction, although access is limited in terms of scope, methods, and groups of individuals. Nevertheless, opportunities have recently increased for AYA facing fertility challenges. In addition to the legal framework outlined, there are other components relevant from a medical perspective. The first to be mentioned in this context is interdisciplinary collaboration between oncologists and reproductive health professionals. In this regard, AYA have a broad need for information and a need to know and understand the interrelationships of different specialties and the available treatment options. Patient empowerment seems to be of imminent importance, especially for AYA. Comprehensive, easily understandable, and up-to-date information about potential consequences for fertility and the possibilities of and issues with reproductive interventions are vital in order for AYA to provide truly informed consent in the face of upcoming cancer treatments. Thus, it is clear that AYA have a great need for adequate education, particularly in the area of germ cell preservation. Digital advances should be harnessed for knowledge transfer, education, and awareness. The provision of information via the various digital channels should serve to empower patients and may inform relatives, families or interested persons. However, individually tailored medical information and discourse is mandatory and cannot be substituted.

## Figures and Tables

**Table 1:** List of AYA organizations.

AYA Organization Categories	Examples
Local advocacy and support organizations	Cancer Fight Club [28] (Canada), Ulman Cancer Fund for Young Adults [29] (USA), Shine [30] (UK), Canteen [31] (Australia), etc.
Organizations for specific AYA	Pink Pearl [32] (young women), Sharsheret [33] (Jewish AYA), Hope for Two [34] (pregnant AYA), etc.
Organizations for specific AYA cancer types	YSC [35] (breast cancer), Tigerlily Foundation [36]. (breast cancer), Testicular Cancer Society [37], National Ovarian Cancer Coalition [38], etc.
Research and education organizations	SAYAO [39], Smart Patients [40], Ovarian Cancer Research Alliance [41], etc.
Web-based social-media platforms or apps	Stupid Cancer [42], Stop Cancer [43], Cancerversity [44] (for young women of color), GRYT Health Cancer Community [45] (Application), Young Adult Cancer Connection [46]

## Data Availability

The data presented in this study are available in this article.
